# Development and Implementation of a Healthcare Database Analysis Course for Graduate Students

**DOI:** 10.3390/pharmacy10050119

**Published:** 2022-09-22

**Authors:** David R. Axon

**Affiliations:** Department of Pharmacy Practice & Science, R. Ken Coit College of Pharmacy, The University of Arizona, 1295 N. Martin Ave., Tucson, AZ 85721, USA; axon@pharmacy.arizona.edu; Tel.: +1-5206-215-961

**Keywords:** graduate pharmacy education, healthcare databases, database analysis, health economics and outcomes research, real-world data

## Abstract

There emerged a need to develop and implement a new healthcare database analysis course for Health Economics and Outcomes Research (HEOR) graduate students, which would allow students to apply their biostatistics and study design skills to answer healthcare-related research questions using large datasets. This communication establishes the need for this course, describes how the course was conceptualized, provides an overview of the course content, course cohort, and course outcomes, and discusses lessons learned from this process. This course was developed to meet the need of HEOR graduates to perform real-world data studies. The course required students to conceptualize a study, apply their data analysis skills to analyze the data, and develop their scientific writing skills by preparing a conference abstract and research report that should be submitted for publication. Lessons learned include focusing more on developing advanced research methodologies and less time on preparing dissemination materials, which can instead be done in subsequent courses or for independent study credit.

## 1. Introduction

Curriculum review may be defined as “a critical examination of academic programs for the purpose of optimizing student learning experiences”. The process of curriculum review should be conducted by the faculty who teach in the program [1]. The field of Health Economics and Outcomes Research (HEOR), like many others, is rapidly evolving [2]. It is therefore important that the required courses offered in HEOR graduate programs are reviewed periodically to ensure they provide a high-quality and relevant education for their students. At the University of Arizona R. Ken Coit College of Pharmacy in the United States (US), a new graduate-level course in healthcare database analysis was developed and implemented for the 2021–2022 academic year. Little has been published on the development and implementation of graduate level courses in the field of HEOR in the US, thus this communication aims to address this gap in the literature. The purpose of this communication is to provide an overview of the development and implementation of this new healthcare database analysis course, and to discuss lessons learned from this process. It is hoped that this communication may provide insight or inspiration to faculty when reviewing their program curriculums, and developing or revising similar courses.

## 2. Establishing the Need for the Course

HEOR graduate programs in the US typically require coursework in the areas of biostatistics, epidemiology, research methodology, Pharmacoeconomics, clinical outcomes assessment, real-world data analysis, systematic reviews and meta-analysis, healthcare policy and management, among others [3]. The Health and Pharmaceutical Outcomes (HPO) graduate program at the University of Arizona R. Ken Coit College of Pharmacy offers Master of Science (MS) and Doctor of Philosophy (PhD) degrees. Students in the HPO graduate program have a set of required core coursework that they must undertake in the early years of their program. This is enhanced by elective and minor coursework, along with independent research study credits, in their later years [3]. Although several elective statistics courses are available to these students from other colleges, the HPO graduate program did not offer a course in real-world healthcare data analysis specifically dedicated to students in this program. That is, students had opportunities to learn new statistical methods, but they did not have an opportunity to apply these techniques to relevant HEOR data. This represented an important gap in their skillset that needed to be addressed to help better prepare these students for their future careers in HEOR. Feedback from students and alumni indicated a desire to learn more about real-world healthcare data analysis. Furthermore, the International Society for Pharmacoeconomics and Outcomes Research (ISPOR) 2022–2023 top 10 HEOR trends identified real world evidence (RWE) as the top trend as its use continues to grow in healthcare decision-making [2]. There was therefore a need to develop such a course, which would allow students to apply their statistics skills to answer healthcare-related research questions using large datasets.

## 3. Conceptualizing the Course

This new course was conceptualized around the goal of providing HPO graduate students with an opportunity to apply the research and statistical skills they had developed in their earlier coursework to answer a novel healthcare question of their choice using large datasets. This course was designed to be offered to students enrolled in the HPO graduate program after they had completed pre-requisite coursework in biostatistics and research methods. Students were therefore expected to be familiar with the concept of designing a study, identifying a data source with relevant variables, importing datasets into a statistical analysis software such as SAS (SAS Institute Inc., Cary, NC, USA), developing and quality checking their statistical coding for their study cohort and variables, and conducting categorical, linear regression, and logistic regression analyses, as appropriate. The course was not intended to teach students statistical methods much beyond what they had already learned in their biostatistics courses, since there are other courses available to fulfill such a purpose. However, students were taught any analytical methods specific to the dataset used in their projects that they were unfamiliar with, such as maintaining cluster and strata in complex survey data and how to use an appropriate weighting variable or conduct a domain analysis.

A second goal of this course was to provide students with an opportunity to disseminate their research. At the time this course was developed, students were learning remotely due to the impact of the COVID-19 pandemic. Consequently, students had not received much in-person mentorship for writing research papers or had an opportunity to present their research at in-person events. This course therefore was designed to provide an opportunity for students to present their research proposal and research findings to their classmates, to prepare and submit an abstract of their work to a national healthcare conference, and to prepare and submit a research report of their project to a peer reviewed journal for publication.

This three credit-hour course was designed to be a small group workshop that met for three hours each week during the semester. In order to participate in this course, students had to be enrolled in the HPO graduate program at the University of Arizona R. Ken Coit College of Pharmacy. The course was planned to be an elective course in the first year, with the possibility of becoming a required HPO graduate course and offered as an elective course to students in other relevant programs in future. The course had a semi-structured design, whereby there was a main task to accomplish each week, yet flexible so that additional time could be spent in areas where it was needed, and less time spent on areas where it was not needed. The course was not designed to have long lectures or copious study notes. Rather, the course was designed to be a “hands-on” experience where students used the class time to work on their projects. Students were expected to come prepared to class and to conduct additional work outside of class as necessary. Course time was predominantly used as an opportunity for students to interact with their classmates and with the course instructor, where they had an opportunity to discuss their ideas for their project, develop and seek feedback on their statistical coding, and seek feedback on their written work. Each student was required to have their own project, although in a spirit of collaboration, students had the option of working with other students in the class (or with external collaborators) as desired to complete their project.

## 4. Description of the Course

The purpose of this course was to gain an understanding of different healthcare data sources and use one of these data sources (Medical Expenditure Panel Survey; MEPS) [4] to develop and answer a research question. Each student was required to present their study to the class, submit an abstract for presentation at a major healthcare conference, and submit a research report for publication in a high-quality peer-reviewed journal. The specific learning objectives for the course are summarized in Table 1.

**Figure 1:**
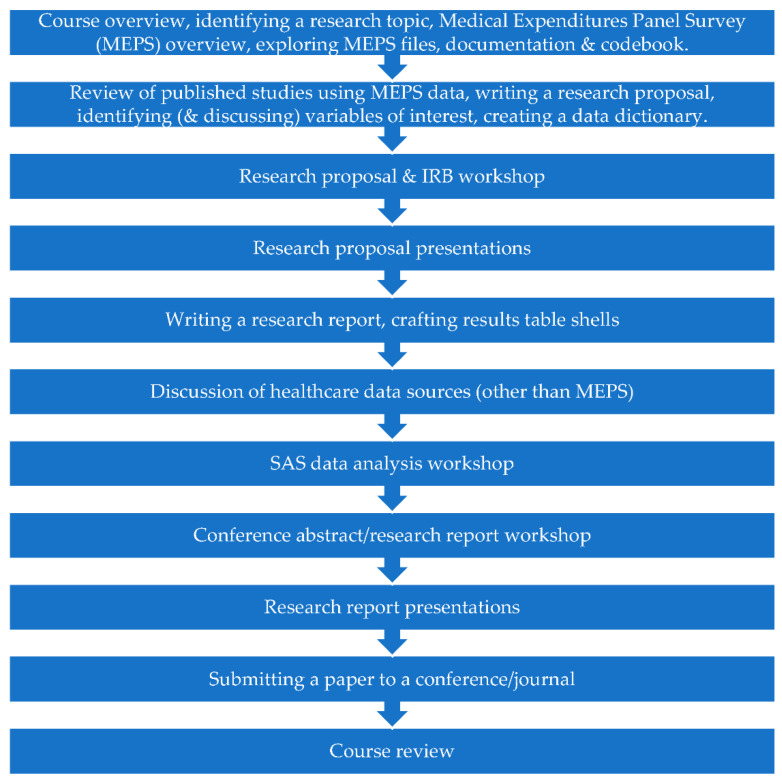
Topics covered in the healthcare database analysis graduate course.

An overview of course activities are provided in Figure 1. The first class had three main activities. First, students were provided with an overview of the course. Second, students were given guidance on identifying a research project that met the FINER (Feasible, Interesting, Novel, Ethical, Relevant) criteria [5]. Third, students were introduced to the Medical Expenditure Panel Survey (MEPS) dataset that they would use for their project. This included an exploration of the publicly available files, documentation, and codebook [6,7].

In the second class, students discussed an example retrospective database study that used MEPS data that they had been assigned to review before class. Students were also asked to identify another study that used MEPS data that was of interest to them, and to discuss the methodology and findings with their peers. Students were tasked with outlining their research proposal, identifying variables that may be of interest to them in the MEPS codebook, and creating a data dictionary for their own study. Students were asked to continue working on their research proposal before the third class.

The third class was a research proposal and IRB application workshop, where students were able to seek feedback from the instructor and incorporate suggested edits into their research proposal and IRB application. Students were instructed that their research proposal should be approximately two single-spaced pages in length, and include a problem statement, specific aims with hypotheses, and proposed methods. The proposal should be supplemented with a data dictionary. Students were also advised that their proposal should be based on the literature, and that they would first need conduct a literature review to identify relevant articles and establish the need for their study.

In the fourth class, students presented their research proposals. This required students to prepare a brief (5–10 min) oral presentation of their research proposal to present to the class. Students were advised they could use slides or any other materials to assist, but this was not required. At the end of each presentation, the instructor and other students in the course offered suggestions to improve the proposal. By the end of this week, students were required to submit their final proposal to the course instructor and to submit their IRB application to the IRB for review. Anticipating that IRB approval would likely take a few weeks, the next two weeks of the course were used to help students begin preparing to disseminate their research, and to discuss other aspects of health care database analysis.

To this end, class five was used to discuss how to write a research report whereby students prepared an outline skeleton of how they anticipated their research report would look. Students also created table shells to enter their results in later. Students also began to explore possible conferences and journals that might be suitable avenues to disseminate their work.

For class six, students were assigned a healthcare data source other than MEPS that was of interest to them and asked to prepare a brief (5–10 min) oral presentation to present to the class. This provided students with an opportunity to investigate a different data set that was of interest to them and share this knowledge with their peers.

By class seven, IRB approval had been obtained for all projects and initiation of developing the statistical analysis code could begin. In total, six classes of this course (classes seven through 12) were allocated to the development of statistical code, data analysis, interpretation of results, and creation of results tables. Students were asked to come prepared with access to SAS and the relevant data and output files.

In class 13, students participated in a workshop to receive feedback on drafts of their conference abstract. Students were tasked with identifying the conference they wished to present at, and to draft the abstract according to author instructions for review in class.

In class 14, students presented the findings of their research. Students were asked to prepare a 15-min oral presentation of their research project to present in class, with an additional 5 min to answer questions. Presentation materials (e.g., slides) were expected to be shared with the instructor and other students ahead of the presentation. Students were also expected to offer feedback to other students for their projects. After these presentations, students were expected to revise their conference abstract and research paper in preparation for final review and submission in class 15.

In class 15, students participated in a final workshop to receive further feedback on their conference abstracts and research reports, and to receive assistance submitting them if they were ready.

Students were graded for their effort in course, which included coming prepared to class, having completed necessary assignments, and actively participating in class activities/discussions (10%), the quality of their research project proposal (20%), obtaining IRB approval (10%), submitting an abstract to a major healthcare conference (10%), and submitting a research report of suitable quality for submission to a high-quality peer-reviewed journal before the end of the semester (50%). A score of 90% was required to earn an ‘A’ grade, with each letter grade dropping by 10%.

## 5. Description of Course Cohort

The first cohort of students in this course consisted of three second-year masters students and one second year PhD student who enrolled in the course for credit, and one additional third-year PhD student who audited the course (i.e., attended the course when possible but did not receive course credit). Three of the students typically attended class in-person, while two typically attended remotely. Two of the students worked together on two projects (one student leading each project and the other supporting), while the remaining three students worked independently. This was the first time any of these students had conducted a research project using real-world data.

## 6. Course Outcomes

At the end of the course, all five students had prepared and submitted an abstract of their work to a national conference, which were all accepted for presentation. However, although all students prepared a research report of their project by the end of the course, only one was submitted for consideration in a peer reviewed journal by the end of the course. The remaining four papers required additional work before they were considered ready to be submitted to a journal. At the time of writing, all papers have been submitted, and in some cases resubmitted, to journals for publication, and one of these papers has already been accepted and published [8].

## 7. Lessons Learned

**Figure 2:**
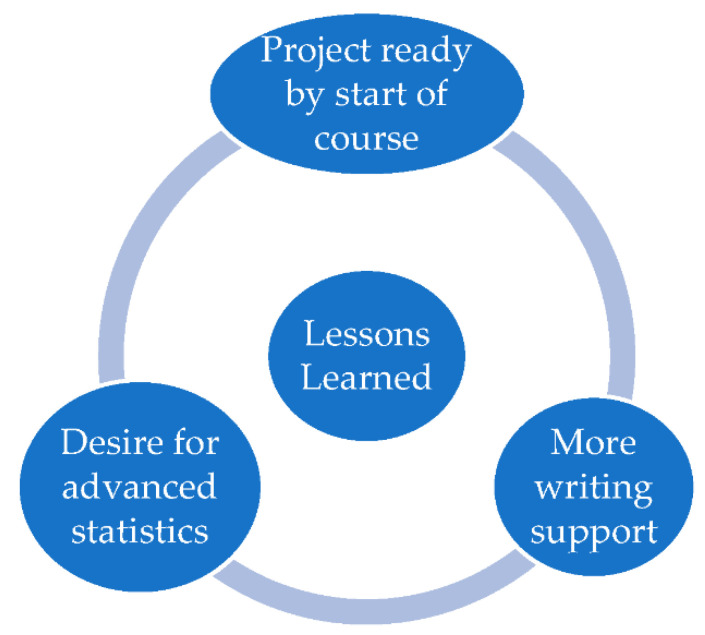
Summary of Lessons Learned.

There were three main lessons learned from teaching this new course which may be useful for future iterations of this course, and to other instructors thinking of developing a similar course at their institution (Figure 2).

The first lesson learned was the importance of encouraging students to have (at least an idea of) a research question in mind for their project at the start of the semester. This was important to ensure that the limited course time available during the 15-week course could be spent productively. Students who came prepared to the course with an idea for their project were able to begin writing their protocol and obtaining IRB approval in a more expeditious manner that those who needed to spend the first few weeks conceptualizing their study. Nevertheless, with a small class size, it was possible for the instructor to ensure all students had a feasible project to conduct in a timely manner.

The second lesson learned was that students did not seem particularly challenged by the statistical analysis for their project. Although the intent was for students to apply the statistical skills they had developed in previous coursework, there appeared to be an interest and desire for learning more advanced techniques, rather than simply applying the skills they had already obtained to a new dataset. Future iterations of this course should therefore focus on providing additional opportunities to learn more advanced statistical techniques as part of the course.

The third lesson learned was that students needed more support with their written work than originally imagined. This was particularly the case with students whose English was not their first language, and students who had less experience writing in a scientific style. For this cohort, it was possible to spend additional course time providing mentorship to students on their written work because the amount of time required to develop and run their statistical code was less than anticipated. For future iterations of this course, it may be worthwhile removing the requirement for a submission-ready research report and allowing additional time to study advanced analytical techniques. A separate course, or independent study credit, could be offered to support students with their scientific writing as needed.

## 8. Conclusions

This communication provides insight into how a new graduate level healthcare database analysis course for HEOR students was conceptualized, developed, and implemented. This communication also describes the course outcomes and and offers suggestions on how the course could be improved. The lessons learned from this course (importance of having a research question in mind for the start of the course, providing additional opportunities to learn more advanced statistical techniques, and providing more writing support) may be helpful to instructors thinking of developing a similar course at their institution.

## Figures and Tables

**Table 1:** Learning objectives for the healthcare database analysis graduate course.

By the end of this course the student will be able to:
Identify a research gap based on contemporary literature.
Formulate a research question and testable hypotheses.
Identify and extract relevant variables and data to answer their research question.
Prepare and submit an application for human subject research.
Present their research proposal and research findings to their peers.
Work collaboratively and offer feedback to their peers.
Appreciate the structure and attributes of complex survey data such as MEPS.
Compare and contrast different healthcare data sources.
Create analytical code using SAS to analyze data.
Interpret output from SAS.
Prepare and submit a research abstract to a major healthcare conference.
Prepare and submit a research report to a peer-reviewed journal.

## Data Availability

Not applicable.
